# Motor Training Increases the Stability of Activation Patterns in the Primary Motor Cortex

**DOI:** 10.1371/journal.pone.0053555

**Published:** 2013-01-07

**Authors:** Yi Huang, Zonglei Zhen, Yiying Song, Qi Zhu, Song Wang, Jia Liu

**Affiliations:** 1 State Key Laboratory of Cognitive Neuroscience and Learning, Beijing Normal University, Beijing, China; 2 Institute of Psychology, Chinese Academy of Sciences, Beijing, China; University of Maryland, College Park, United States of America

## Abstract

Learning to be skillful is an endowed talent of humans, but neural mechanisms underlying behavioral improvement remain largely unknown. Some studies have reported that the mean magnitude of neural activation is increased after learning, whereas others have instead shown decreased activation. In this study, we used functional magnetic resonance imaging (fMRI) to investigate learning-induced changes in the neural activation in the human brain with a classic motor training task. Specifically, instead of comparing the mean magnitudes of activation before and after training, we analyzed the learning-induced changes in multi-voxel spatial patterns of neural activation. We observed that the stability of the activation patterns, or the similarity of the activation patterns between the even and odd runs of the fMRI scans, was significantly increased in the primary motor cortex (M1) after training. By contrast, the mean magnitude of neural activation remained unchanged. Therefore, our study suggests that learning shapes the brain by increasing the stability of the activation patterns, therefore providing a new perspective in understanding the neural mechanisms underlying learning.

## Introduction

From using chopsticks to playing piano, a certain amount of training is required. However, functional magnetic resonance imaging (fMRI) studies provide contradicting findings on the neural mechanisms underlying learning. After training, the mean magnitude of neural activation in a region of interest can be either increased [1], [2], [3], [4], [5], [6], [7], decreased [2], [3], [6], [8], [9], [10], [11] or even unchanged [12]. We argue that one possible interpretation of these inconsistent findings is that learning-induced changes are not homogenous in that the activation of some neurons is increased and the activation of others is decreased (for a review, see [13]). Accordingly, fMRI studies based on the mean magnitudes of neural activation averaged across voxels may show inconsistent findings.

Neurophysiological studies on monkeys have unequivocally demonstrated the heterogeneous nature of changes in neuronal activation after training. In these studies, training generally increases the selectivity of neurons to trained stimuli or tasks, but the increased selectivity is achieved in different manners. One method is to sharpen the tuning curve of either the least responsive neurons [14], [15], [16] or the most informative neurons [17], [18]. Another method is to modify the magnitude of neuronal activation by either decreasing the responses for untrained stimuli [19] or increasing the responses for trained stimuli [14], [15], [17]. Therefore, when learning-induced neuronal changes are examined at the population level, contradictory results that are similar to those observed in the fMRI studies are observed. That is, the neural activation is either decreased [20], [21], increased [22], [23], [24] or unchanged [25].

In this study, we proposed a new approach to investigate the neural mechanisms underlying learning at a finer scale using a multi-voxel pattern analysis (MVPA) [26]. This new approach is derived from two previous findings. First, learning modifies the tuning of activation patterns for task-relevant features [27], [28], [29], [30]. Second, MVPA is suitable for revealing heterogeneous neural activation, such as the orientation map in the primary visual cortex [31]. Therefore, if motor training leads to heterogeneous changes in the primary motor cortex (M1), we expect significant changes in the activation patterns for trained (versus untrained) finger-tapping movements, even without significant changes in the mean magnitudes.

## Methods

### Participants

Ten college students (aged 21–30 years; 4 males) were recruited from Beijing Normal University, Beijing, China. All participants were right-handed, and none were professional typists or musicians. The experimental protocol was approved by the Institutional Review Board of Beijing Normal University. Written informed consent was obtained from each participant before the experiment.

### Behavior Training

A classic finger-tapping task [1] was used in the behavioral motor training. In a finger-tapping training session, the participants were instructed to perform a tapping movement with the fingers in a specific order (e.g., from little finger, to index finger, to ring finger, and to middle finger) as accurately and quickly as possible in 30 sec with the left hand (i.e., the non-dominant hand). No visual feedback was provided throughout the session. The entire session was videotaped, and two observers who were unaware of the objective of this study independently calculated the number of correctly completed sequences in the session by watching the video recordings. The number of correctly completed sequences was used as an index for motor performance in the session. Each participant completed forty sessions per day for five consecutive days, which generated 200 total sessions for each participant. The participants took a short break between sessions and a long break between the first and second half of 40 sessions. There were two tapping sequences (Order one: 4(little), 1(index), 3(ring), and 2 (middle); Order two: 2 3 1 4) in which one order was the to-be-trained sequence and the other was the untrained sequence. One-half of the participants were assigned to the first tapping sequence, and the other half were assigned to the second sequence. The participants’ performance for both tapping sequences was examined before and after training, whereas only one sequence was trained during the training period.

### fMRI Experiment

**Figure 1:**
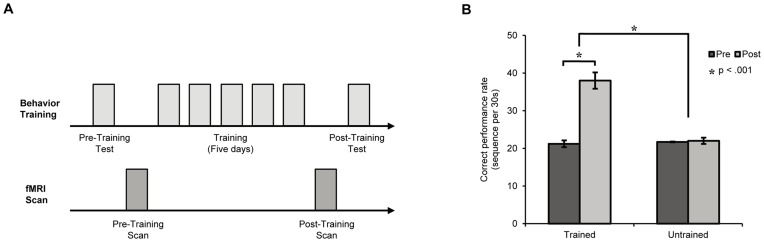
Experimental procedure and behavioral results. A) Participants were instructed to practice sequential finger-tapping movements for five consecutive days. Pre- and post-training behavioral tests were conducted to measure the improvement of behavioral performance in finger tapping. FMRI scans were conducted before and after motor training to examine the learning-induced changes in neural activation. B) Behavioral performance was measured as the number of correct sequential finger-tapping movements per 30 sec for both the trained and untrained sequences occurring before and after motor training. The error bars indicate ±1 standard error of the mean (S.E.M.).

There were two fMRI scan sessions for each participant that were performed before and after the behavioral training (Figure 1A). Each fMRI session consisted of (1) a one blocked-design localizer run and (2) seven slow event-related-design experimental runs.

In the localizer scan, the participants were instructed to randomly tap their fingers to localize the M1. In a block, the instruction, either “left hand” or “right hand,” was displayed on the screen for 15 sec. The participants performed random finger-tapping movements with the hand that corresponded to the instruction until the instruction disappeared. The order of the blocks for the left- and right-hand movements were randomly mixed and counterbalanced with a 15 sec rest (i.e., no tapping) between each tapping block. There were four blocks for each hand movement.

In the experimental scan, the participants were instructed to perform finger tapping movements using either the trained sequence or the untrained sequence with the left hand (i.e., the trained hand). In a trial, the order of the sequence (e.g., 4, 1, 3, 2) was presented on the center of the screen for 750 ms followed by flickering dots at 4 Hz. The participants tapped their fingers in a sequence based on the instructions for the rhythm of the flickering dots (i.e., one tap at the presence of one flickering dot). Because the participants tapped during both the trained and untrained sequences at identical speeds, the total amount of motor movements was matched for the two sequences. Therefore, the differences observed between the performances for the two sequences during the fMRI scan cannot be accounted for by the difference in either the speed of tapping or the amount of motor movements. The trials consisting of the trained and untrained sequences were randomly mixed. Between each trial, a blank screen with a fixation point jittered between 16.5 sec and 19.5 sec to ensure that the hemodynamic activities returned to baseline. There were nine trials for the trained and untrained sequences in each run, which generated sixty-three total trials per condition (i.e., seven total runs).

### MRI Acquisition

MRI data were acquired on a Siemens 3T Trio scanner (MAGENTOM Trio, a Tim system) with a 12-channel phased-array head coil at the BNU Imaging Center for Brain Research, Beijing, China. T2*-weighted functional images were acquired with a gradient-echo, echo-planar imaging (EPI) sequence (TR = 1.5 sec, TE = 30 ms, FA = 90 degrees, FOV = 200×200 mm, matrix = 64×64, number of slices = 25, and voxel size = 3×3×4 mm). T1-weighted structure images were also collected with a magnetization-prepared rapid gradient-echo (MPRAGE) sequence (TR/TE/TI = 2.53 sec/3.45 ms/1.1 sec, FA = 7 degrees, voxel size = 1×1×1 mm) for each participant.

### fMRI Data Analysis

Functional data were analyzed with the fMRI Expert Analysis Tool (FEAT) implemented within FMRIB’s Software Library (FSL) (http://www.fmrib.ox.ac.uk) and in-house MATLAB codes. Data preprocessing was applied with default options in FEAT, including head motion correction with a six-parameter affine transformation implemented in MCFLIRT (Motion Correction using FMRIB's Linear Image Registration Tool), brain extraction, spatial smoothing on a Gaussian kernel (5 mm full width at half maximum) and a high-pass temporal filter (a Gaussian-weighted running line filtering with a cutoff of 100 sec). Time-series statistical analyses were conducted using FILM (FMRIB's Improved Linear Model) with local autocorrelation corrections. Each run was modeled separately for each participant.

The localizer runs were modeled by a boxcar convolved with a gamma hemodynamic response function and its temporal derivative. Each participant’s motor region specific to left-hand finger tapping was localized with the contrast of left-hand tapping versus right-hand tapping; the reverse contrast was used to define the motor region for right-hand finger tapping.

For the experimental runs, a gamma hemodynamic response function and its temporal derivative were used to model each slow event. Each participant’s BOLD response to each sequence was defined with the contrast of the trained sequence versus the fixation and the contrast of the untrained sequence versus the fixation respectively. In addition, each participant’s functional data were registered to his/her high-resolution structure images and then to the standard space images (Montreal Neurological Institute MNI-152 template) using FLIRT (FMRIB's Linear Image Registration Tool) of FSL.

We used M1 as an ROI for further ROI-based analyses because it is the most studied region in motor-sequence training [1], [32] and because it provides fine-tuned representations for finger movements [33], [34], [35]. Specifically, M1 in the right hemisphere (corresponding to the left hand) was defined by intersecting the functional activation (p<10^−12^, uncorrected) in the localizer run and the anatomic M1 label derived from maximum probabilistic maps (thresholded at 25%) of the Juelich Histological Atlas [36] implemented in FSL. In an identical manner, M1 in the left hemisphere served as a control region but with the contrast of the tapping movement of the right hand versus that of the left hand.

To calculate the learning-induced changes in the activation patterns for the trained sequence, MVPA was performed on the beta value across voxels in M1. First, all experimental runs were divided into even and odd runs, and the beta value of each voxel was averaged across the even and odd runs for each tapping sequence. Second, the correlations between the activation pattern based on the averaged beta values in the even and odd runs were calculated. Specifically, the within-sequence correlation was calculated between the even and odd runs for the trained sequence, whereas the between-sequence correlation was calculated in an identical manner but between the trained and untrained sequences. The difference between the within- and between-sequence correlations indicated the similarity in the spatial patterns of neural activation between the even and odd runs that were specific to the trained sequence, or the stability of the activation pattern for the trained sequence. Finally, the training effect was measured as the change in stability of the activation pattern for the trained sequence between the post- and pre-training scans. In addition, the training effect for the untrained sequence was calculated in an identical manner.

In addition to the ROI-based analysis, we also performed a searchlight analysis across the entire brain to examine learning-induced changes in activation patterns in the brain. In the analysis, the searchlight was a roving small ROI across the entire brain volume [37]; the size of the cubic searchlight was 7×7×7 voxels, and in turn, every voxel of the brain was the center of the searchlight. The training effect was calculated in an identical manner as that in the ROI-based analysis, and a two-tailed *t*-test between the trained and untrained sequences was calculated to generate a *t*-map of the entire brain.

Finally, we used a traditional univariate method to measure learning-induced changes based on the mean magnitudes of neural activation. Initially, the beta values of all voxels within an ROI were extracted for each condition, run, and participant. Subsequently, the beta values were averaged across voxels and runs for each condition and participant. Finally, the training effect was calculated as the difference between the mean magnitude in the post-training scan and that in the pre-training scan.

## Results

### Training Improves Behavioral Performance on Finger Tapping

Before training, the mean speed of correct finger tapping was 21.2 and 21.7 times per 30 sec for the to-be-trained and untrained sequences, respectively. After training for five consecutive days, the speed of the trained sequence was 38.0 times per 30 sec, whereas the speed of the untrained sequence remained unchanged (22.0 times per 30 sec) (Figure 1B). A two-way ANOVA of tapping sequence (trained versus untrained) by training (pre- versus post-training) showed a significant main effect of training (F(1, 9) = 90.5, p<0.001) and a significant main effect of tapping sequence (F(1, 9) = 70.1, p<0.001). Importantly, the two-way interaction of tapping sequence by training was significant (F(1, 9) = 39.7, p<0.001), which indicated that the improved behavioral performance was specific to the trained sequence. *Post hoc t*-tests confirmed this observation, with the performance on the trained sequence being significantly higher after training than before training (t(9) = 8.7, p<0.001, Cohen’s d = 3.30) and no significant change for the untrained sequence (t(9) <1, Cohen’s d = 0.09). An ANOVA on accuracy revealed a similar pattern, with a significant two-way interaction of tapping sequence by training (F(1, 9) = 20.6, p = 0.001). *Post hoc t*-tests further revealed that there was no significant difference in accuracy between two sequences before the training (t(9) <1, Cohen’s d = −0.21), whereas the accuracy for tapping the trained sequence was significantly higher than that for the untrained after the training (t(9) = 7.0, p<0.001, Cohen’s d = 2.54). Next, we investigated the neural mechanisms underlying the learning-induced behavioral changes.

### Motor Training Improves the Stability of Activation Patterns in M1

**Figure 2:**
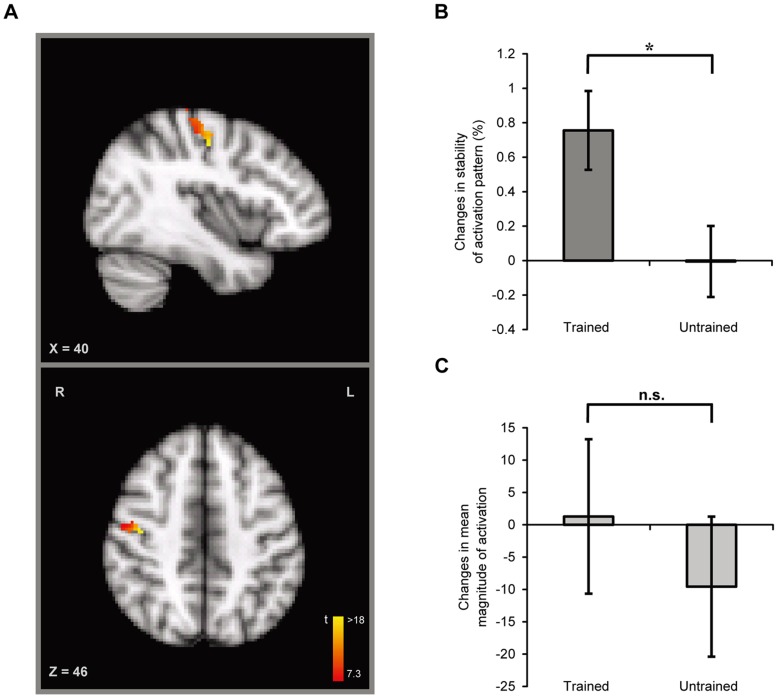
ROI-based Analyses. A) The right primary motor area (M1) that corresponds to the trained left hand from a typical participant (MNI coordinates: x = 40, y = −14, z = 46; t value = 18.3). M1 was defined as the intersection between the functional activation in the localizer scan and the anatomic M1 label in the Juelich Histological Atlas. B) Learning-induced changes in the stability of activation patterns in the M1 for both trained and untrained sequences. C) Learning-induced changes in the mean magnitudes of neural activation for both the trained and untrained sequences. The error bars indicate ±1 S.E.M. An asterisk indicates p<0.05.

The primary motor cortex (M1) involved in finger tapping movements was defined as the intersection between the functional activation induced by random finger-tapping movements of the trained hand (i.e., the left hand) in the localizer scan and the anatomic M1 label in the Juelich Histological Atlas (the MNI coordinates of the peak voxel in the right M1: x = 36, y = −30, z = 62) (Figure 2A).

To examine the learning-induced changes in M1, we calculated the similarity of activation patterns between the even and odd runs for the trained sequence, which indicates the stability of activation patterns (see Methods). The training effect was thus defined as changes in the stability of activation patterns between the post- and pre-training scans. We observed that in the right M1, which corresponds to the trained hand, motor training significantly increased the stability of the activation pattern induced by the trained sequence (t(9) = 3.3, p = 0.01, Cohen’s d = 1.05) and not the untrained sequence (t(9) <1, Cohen’s d = −0.01) (Figure 2B). Notably, the learning-induced change in the stability of the activation patterns for the trained sequence was significantly larger than that for the untrained sequence (t(9) = 2.7, p = 0.02, Cohen’s d = 0.86).

By contrast, in the left M1, which corresponds to the untrained hand (MNI coordinates of the peak voxel in the left M1: x = −42, y = −20, z = 56), we did not observe learning-induced changes in the stability of the activation patterns for either the trained (t(9) <1, Cohen’s d = −0.05) or untrained sequences (t(9) = 1.0, p = 0.33, Cohen’s d = 0.36). Moreover, there was no significant difference in the training effect between the trained and untrained sequences (t (9) <1, Cohen’s d = −0.22).

**Figure 3:**
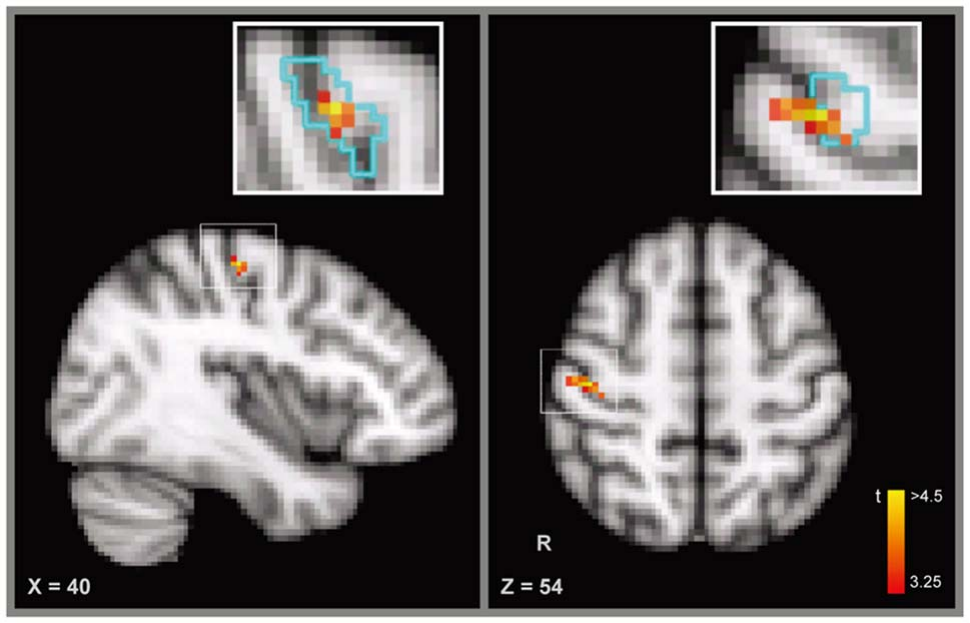
Searchlight Analyses. A cluster of voxels in the right M1 show the increased stability of the activation pattern for the trained sequence (MNI coordinates: x = 52; y = −18; z = 58; t value = 4.76). The inset figure shows the overlap between the cluster identified by the searchlight analysis and the M1 identified in the localizer scan (outlined in cyan).

To further examine whether the learning-induced change in the stability of activation patterns was specific to the right M1, we performed a searchlight analysis across the entire brain. The searchlight analysis revealed a cluster of voxels in the right M1 showing that the increase in the stability of the activation pattern was specific to the trained sequence after training (p<0.01; uncorrected) (MNI coordinates of the peak voxel in the cluster: x = 52, y = −18, z = 58) (Figure 3). Not surprisingly, the cluster identified by the searchlight analysis was partially overlapped with M1 that was used in the ROI-based analysis (percentage of overlap: 39%; the distance between the peak voxel in the cluster and that in M1∶1.33 cm; Figure 3). No other continuous cluster (cluster size >20 voxels) was observed in other cortical regions of the brain. Of note, with a smaller size (5×5×5 voxels) and the sphere-shape searchlight, we observed the similar result.

Finally, we examined whether the behavioral motor training changed the mean magnitude of neural activation in M1. The training effect was defined as changes in the mean magnitude between the post- and pre-training scans. No learning-induced changes in the mean magnitude were observed for either the trained (t(9) <1, Cohen’s d = 0.04) or untrained sequences (t(9) <1, Cohen’s d = −0.30) (Figure 2C). Moreover, there was no significant difference in the training effect between the trained and untrained sequences (t(9) = 1.9, p = 0.09, Cohen’s d = 0.62).

## Discussion

In this study, we used fMRI to investigate whether motor training shaped the stability of activation patterns in the primary motor cortex. Behaviorally, we showed that the performance on sequential finger tapping movements was significantly improved after the training, and the training effect was specific to the trained sequence. Neurally, both ROI-based MVPA and the searchlight analysis revealed that the stability of activation patterns, or the similarity of the activation patterns between the even and odd runs, significantly increased for the trained sequence but not for the untrained sequence. In addition, the learning-induced change was only observed in M1 that corresponded to the trained hand but not the untrained hand. By contrast, we did not observe significant changes in the mean magnitudes of neural activation in M1 after motor training. In short, our study provides direct evidence showing learning-induced changes in activation patterns, even without detectable changes in the mean magnitudes of neural activation.

The finding that motor training shaped activation patterns in M1 extends previous studies based on mean magnitudes of neural activation in motor training tasks. In these studies, both the increased [1], [5], [38], [39] and decreased neural activations [1], [32] in M1 were reported because of motor training. Two stages of learning processes have thus been proposed to address these apparently inconsistent findings [32], [40]; see also [41]. At the early stage of training, which can be as brief as a few minutes, the mean magnitude of neural activation in M1 is typically decreased. By contrast, when the duration of motor training is extended to several weeks, the mean magnitude of neural activation in M1 is often increased. The duration of motor training in our study (five days) was between the early and late stages; therefore, it is not surprising that we did not observe changes in the mean magnitudes of neural activation in M1. Importantly, our study may provide insight on how motor training shapes M1 between the early and later stages. During this period, motor training may increase the stability of neural representation in the M1 for the trained motor behavior, without showing an evident change in the mean magnitudes of neural activation at the regional (or population) level. The increased pattern stability may lead to better retrieval of motor memory. As a result, the behavioral consistency in tapping, such as trial-by-trial variability in response time, may be improved [42].

Previous studies have shown that the greater stability of activation patterns is associated with conscious (versus unconscious) experiences [43], better memory retrieval [44] and better behavioral performance in face recognition [45]. This close link between pattern stability and behavioral performance suggests that greater pattern stability may serve as a neural marker for a more refined and efficient representation that leads to better behavioral performance. Therefore, the increased pattern stability after training in our study provides a new insight on the underlying mechanism for learning-induced changes in the brain (see also [46]). That is, neural representations for trained stimuli can be established and refined through modulation on pattern stability by training. Importantly, previous studies have shown that neural representations differ in sparseness that stimuli can be represented either by response magnitude of a small number of sharply-tuned neurons (sparse coding) [47], [48], [49] or by concerted activity pattern distributed across a large population of broadly-tuned neurons (population coding) [50]. Accordingly, more stable and refined representations after training might be manifested either as less variability in local neuronal response amplitude or regional average response amplitude [42], [51] or as more similar spatial activation patterns across multiple voxels [46]. Therefore, homogenous and independent changes of neuronal tunings many lead to increased stability of mean response amplitude; in contrast, heterogeneous and interrelated changes of neuronal tunings may result in increased stability of activation patterns. Because representations in the motor cortex are largely in population coding [52], [53], it is not surprising that we observed the increased stability of activation patterns after motor training.

Future fMRI studies with a higher spatial resolution and more scan sessions at multiple time intervals during training may help to illustrate how learning shapes the brain by changing both the mean magnitudes and spatial patterns of neural activation.
